# Effect of Vanadium Content on the Microstructure and Mechanical Properties of IN718 Alloy by Laser Cladding

**DOI:** 10.3390/ma14092362

**Published:** 2021-05-01

**Authors:** Hao Lv, Zhijie Li, Xudong Li, Kun Yang, Fei Li, Hualong Xie

**Affiliations:** 1 Department of Mechanical Engineering and Automation, Northeastern University, Shenyang 110819, China; lvhao@me.neu.edu.cn (H.L.); 1800309@stu.neu.edu.cn (Z.L.); 1800308@stu.neu.edu.cn (X.L.); 2 School of Mechanical Engineering, Southeast University, Nanjing 211189, China; yk1873639934@163.com; 3 Department of Information Science and Engineering, Shenyang University of Technology, Shenyang 110870, China; lifei@sut.edu.cn

**Keywords:** laser cladding, microalloying, vanadium addition, Laves phase, IN718 alloy

## Abstract

Microalloying vanadium can change the segregation state of Nb element in IN718 alloy, reduce the formation of harmful Laves phase and refine the dendritic structure of IN718 alloy during the laser process. Therefore, IN718 alloys with V content from 0.081 to 1.88 wt.% were prepared and evaluated. Metallographic microscopy and scanning electron microscopy were used to observe the corresponding morphology, structure, and distribution of elements. First of all, it was found that the addition of V refines the grain size of IN718 alloy and reduces the primary dendrite arm spacing. Secondly, adding V to IN718 alloy can reduce the porosity of the cladding layer. The elements are uniformly distributed in the cladding layer, and the addition of vanadium reduces the segregation degree of the Nb element, which is conducive to homogenization. In addition, microhardness and residual stress were also investigated. Finally, the addition of vanadium was shown to have no apparent effect on the tensile strength and yield strength but can significantly improve the elongation of IN718 alloy. In conclusion, the microstructure and mechanical properties of IN718 alloy with 0.081 wt.% vanadium content provide a new solution to improve the application level of IN718 alloy in laser cladding.

## 1. Introduction

IN718 alloy is designed for strength, oxidation resistance and good fatigue life at high temperatures of up to 700 °C, in addition to excellent weldability through its relatively slow precipitation kinetics [1,2,3]. Because of its excellent mechanical properties at high temperatures, it has become a widely used material in the fields of aviation, aerospace, nuclear energy, and petroleum [4]. IN718 alloy is an aging-strengthened Ni-Fe-Cr-based deformed high-temperature alloy. During the solidification process, it will produce severe component segregation, which will lead to the generation of Laves phase. The Laves phase is a hard and brittle phase that can cause cracks and lead to crack propagation [5,6]. Therefore, the improvement of Laves phase is significant in the improvement of the microstructure, as well as overall changes in the performance, of IN718 alloy.

**Figure 1:**
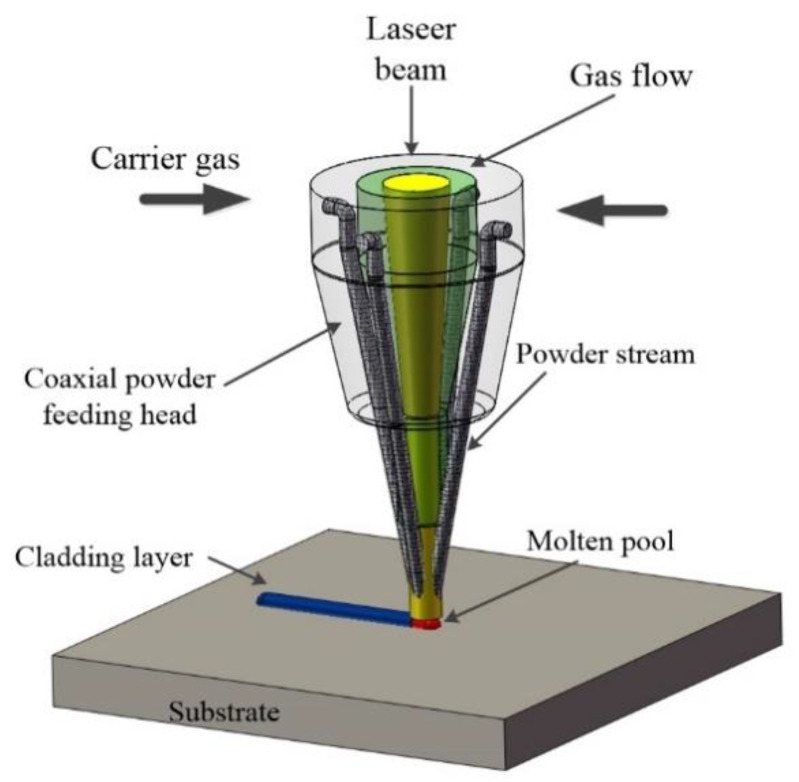
The schematic diagram of DLD in laser cladding.

Laser cladding is a very advanced surface engineering method and has been widely used in surface modification [7]. The high-energy-density laser radiation quickly melts the alloy powder and the surface of the substrate. Direct laser deposition (DLD) involves sending the powder into the molten pool at the same time as the laser is irradiated. The schematic diagram of DLD in laser cladding is shown in Figure 1. Due to the rapid heating and cooling rate of laser cladding, the cladding layer has a dense structure and low porosity.

Many studies have used the addition of different elements to control the degree of segregation of the Laves phase, thereby improving the properties of the IN718 alloy. The addition of Co can cause the solubility of molybdenum in the dendritic nucleus, and the reduction of Mo in the residual liquid. As a result, the Laves phase is restrained while the precipitation of δ-Ni3Nb is promoted [8]. The addition of phosphorus and boron to the IN718 alloy promotes the formation of the bulk Laves phase [9]. The increase in the Al/Ti atomic ratio significantly reduces the degree of element segregation in IN718 alloy during casting [10]. The addition of phosphorus can change the propagation path of cracks from the matrix (Laves phase) around the particles to the interface between the matrix and the particles [11]. Zr can inhibit the precipitation of Laves phase at the grain boundary of K4169 alloy [12].

Vanadium (V) has a high solubility in austenite and is one of the most commonly used and effective strengthening elements for micro-alloyed steel [13]. The effect of vanadium on the solidification and homogenization behavior of IN718 alloy was studied. It is observed that vanadium is uniformly distributed in the dendrite and interdendritic regions, which can effectively prevent the precipitation of the Laves phase [14]. The influence of different contents of vanadium on the microstructure and mechanical properties of IN718 alloy in casting was evaluated. Vanadium changes the microstructure characteristics of the IN718 alloy and affects its mechanical properties, and a relatively compromised vanadium content is obtained [15].

In our previous research [16], the addition of vanadium was shown to have a positive effect on the microstructure uniformity, Lave phase distribution and mechanical properties of the IN718 alloy. Hence, we studied the effects caused by the addition of 0.081–1.88 wt.%, by weight, of vanadium into IN718 alloy on the microstructure and mechanical properties of the laser cladding.

## 2. Materials and Methods

### 2.1. Materials

**Figure 2:**
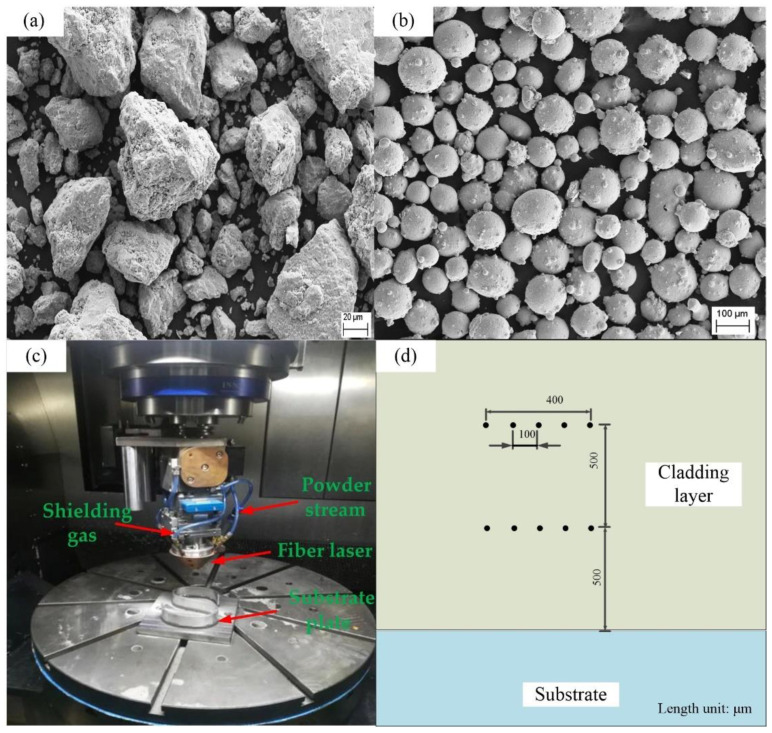
(**a**) Morphology of V powder (1000×), (**b**) morphology of IN718 alloy power (200×), (**c**) SVW80C-3D hybrid additive and subtractive machine center, (**d**) Vickers hardness measurements points [16].

The experimental materials are mainly divided into two types, one is vanadium powder, which is prepared primarily by the atomization and crushing process (ACP), and its shape is irregular (Figure 2a), and the other is IN718 alloy powder, which is mainly produced through the plasma rotating electrode process (PREP). For the spherical powder prepared by the plasma rotating electrode process (Figure 2b), the average diameter of the above two powders is 100 μm. The material used for the substrate is also IN718 alloy, with a size of 100 mm × 100 mm × 8 mm. The nominal composition of the master alloy (in mass fraction, %) is C 0.02, Cr 19.23, Nb 5.00, Ti 1.08, Mo 3.2, Al 0.54, Fe 18.64, balance Ni. IN718 alloys with different amounts of vanadium are shown in Table 1 [14].

### 2.2. Experimental Parameters

Before the experiment, the two powders were kept in a dry box at 150 °C for two hours. The substrate was polished with coarse sandpaper to remove the oxide layer on the surface and, finally, washed with acetone and absolute ethanol to remove the oil stains. The laser cladding processing system is carried out in a composite machining center for adding and reducing materials. The system is mainly composed of a numerical control system, a laser, a powder feeder, a cooler, an air compressor and a high-purity nitrogen generator, as shown in Figure 2c. The length of the laser spot is 3 mm and the width is 1 mm.

In order to ensure that the V powder is evenly mixed in the IN718 powder, the two powders are stirred in a ball mill for 60 min. Each time a different proportion of powder is carried out, the powder in the powder feeder is fully emptied, the purpose of which is to ignore the influence of impurities on the experimental results. For the microstructure, porosity, microhardness, etc., a single-pass experiment was performed, and the cladding layer was cut along the scanning direction. The sample was etched with Kalling’s etchant (40 mL HCl, 40 mL C_2_H_5_OH, 2 g CuCl_2_). A Zeiss ULTRA PLUS Scanning Electron Microscope (SEM, Zeiss, Oberkochen, Germany) with a X-Max 50 Energy Dispersive Spectrum (EDS, Oxford, UK) and an OLYMPUS-OLS4100 Confocal Laser Scanning Microscope (CLSM, OLYMPUS, Tokyo, Japan) observed and measured the microstructure and chemical composition, respectively. The chemical composition of the phase in the molten was characterized by the energy spectrometer with SEM, and the acceleration voltage in EDS analysis is 20 kV. The average size of the primary dendrite spacing (PDS) of the columnar dendrites was calculated using Image-Pro Plus 6.0 software (Ropers Technologies, Sarasota, FL, USA). The microhardness test was performed using the HVS-1000 M Vickers microhardness tester (Ledi Instruments Co., Ltd., Ningbo, China) with a load of 50 gf and a dwell time of 8 s. The cladding layer inscribed at the test points was 0.5 mm away from the substrate, and the measurement was performed every 0.1 mm. The average value of 5 points was taken as the microhardness under the process parameters. The measurement position is shown in Figure 2d.

**Figure 3:**
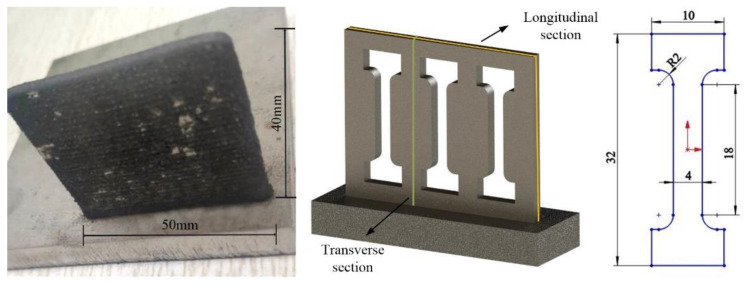
Cutting positions and dimension of the tensile specimens.

In addition, the microhardness method was used to measure the residual stress of the cladding layer [17,18]. The tensile test was performed using an electronic universal material testing machine (WDW-100E, manufactured by Panasonic, Osaka, Japan). The maximum load, crosshead speed and temperature were set to 100 kN, 2 mm/min and 300 K, respectively. In order to ensure the statistical significance of the test results, three sets of tensile specimens were tested under the same parameters. As shown in Figure 3, the tensile specimen was cut from the thin-walled specimen by wire electrical discharge machining (WEDM, Sodick Co., Yokohama, Japan) [19]. The dimensions of the thin-wall specimens were 40 mm (along the laser deposition direction) × 3 mm × 50 mm (along the laser scanning direction). The process parameters were determined according to the previous study [20] and the detailed parameters are listed in Table 2

## 3. Results and Discussion

### 3.1. Effect of Vanadium Content on Microstructure Characteristics of Solidification

**Figure 4:**
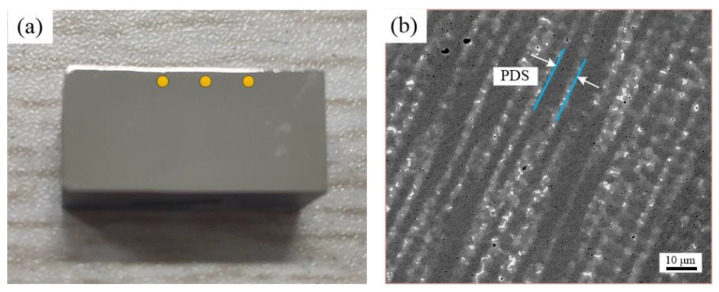
(**a**) Test point location, (**b**) the columnar dendritic morphology of the alloy.

The solidification structure is composed of primary austenite dendrites and interdendritic segregation [21,22]. Three locations were measured to calculate the volume fraction of crystal nuclei and primary dendrite arms spacing (PDS) values, along with the sample in the scanning direction of the cladding. The selected images are typical areas of columnar grains under each alloy, as shown in Figure 4a. At the corresponding position, three different pictures were chosen for each alloy. Figure 4b shows an image of typical columnar crystal regions in each test alloy. In addition, the white area represents the trunk of the dendrite, and the black area represents the interdendritic region.

**Figure 5:**
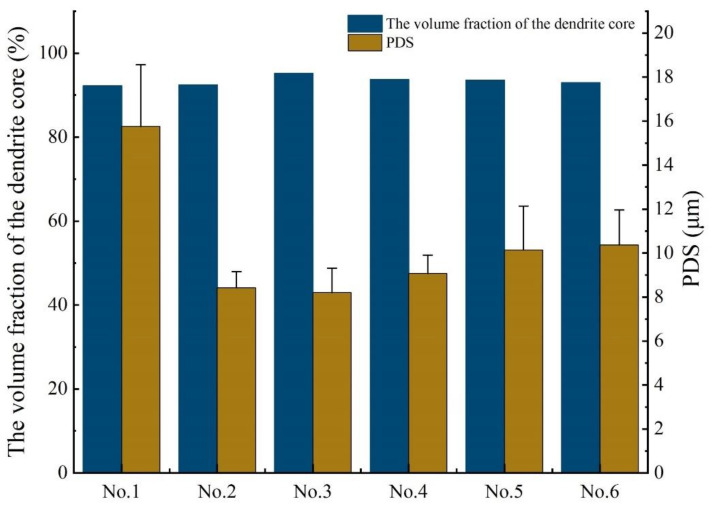
Comparison of the volume fraction of the dendrite core and PDS for all tested alloys.

The results are shown in Figure 5. The results show that the addition of vanadium will increase the volume fraction of the dendrite core, and No. 3 alloy has the largest dendrite core fraction. However, the dendrite core of other alloys has a smaller range in comparison. The measurement of the primary dendrite arm spacing of the columnar dendrite adopts the cutting line method. It can be seen from Figure 5 that the addition of vanadium powder will reduce the primary dendrite arm spacing. In addition, the value of No. 3 alloy is, similarly, the smallest. The addition of vanadium leads to the microstructure refinement of many alloys [23,24,25], which is also believed to make the structure of IN718 alloy more refined.

**Figure 6:**
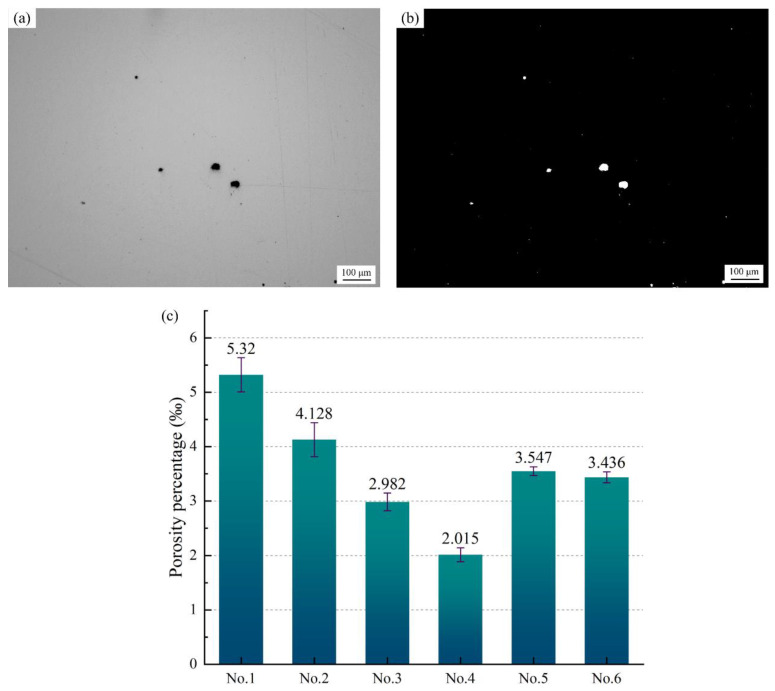
(**a**) Pore distribution of cladding layer, (**b**) pore distribution process after binarization, (**c**) comparison of porosity of different tested alloys.

It is well known that porosity has a significant effect on the quality and reliability of cladding layers. The distribution and size of the pores will significantly affect the various mechanical properties and structural morphology of the cladding layer, such as premature crack formation, low ductility, residual stress and fatigue properties [26,27,28]. Many studies have established a connection between pores and properties in IN718 alloy, and reduced porosity, based on numerical simulations, processing parameters, scanning strategies, cooling rates and other solutions [29]. Figure 6a is the original microscope picture of the cladding layer in different tested alloys. After binarization of image software, the picture of porosity, as shown in Figure 6b, is obtained, from which the porosity is calculated. From Figure 6c, it can be found that there are obvious differences in the porosity of various alloys. The porosity of the IN718 alloy with vanadium added is lower than that of the blank sample. In addition, the porosity of No. 5 alloy and No. 6 alloy are relatively close. Finally, the porosity of No. 4 alloy reached the lowest level. It can be predicted that the mechanical properties of No. 3 alloy and No. 4 alloy are better than those of the blank sample.

### 3.2. Effect of Vanadium Content on Element Segregation

**Figure 7:**
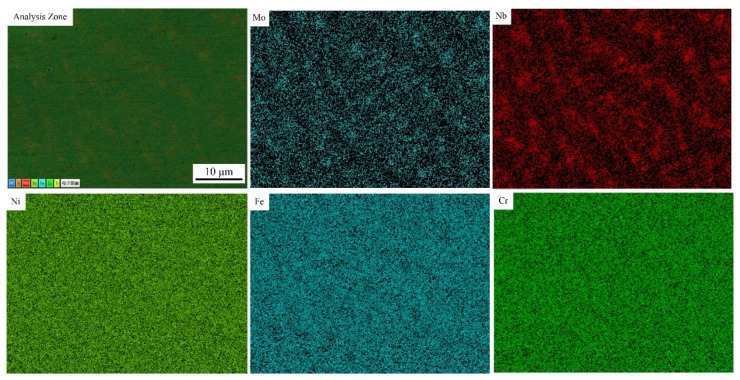
Element distribution in No.1 alloy.

**Figure 8:**
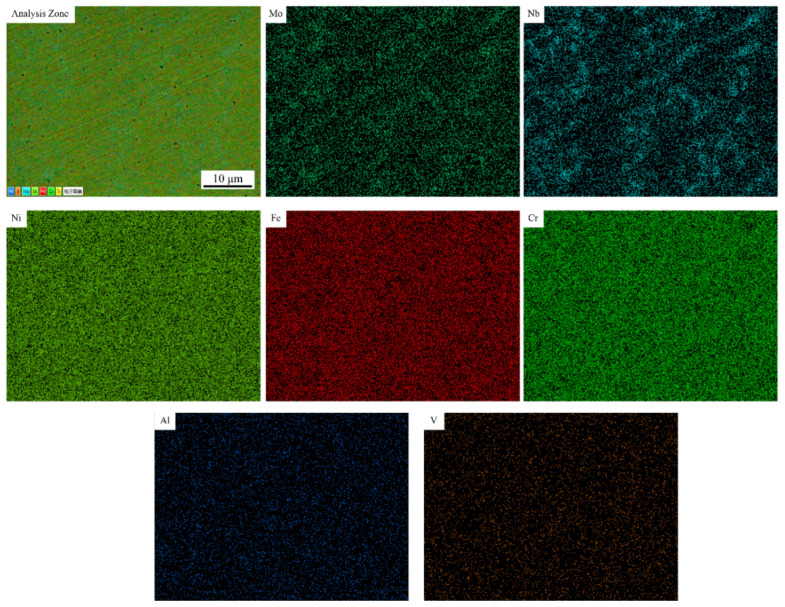
Element distribution in No.3 alloy.

Through surface scanning analysis, the element distribution of the No. 1 alloy and No. 3 alloy were selected to represent randomness. The brighter the color, the higher the concentration, as shown in Figure 7 and Figure 8. In the No. 1 alloy, Mo and Nb are mainly distributed in the interdendritic region and are positive segregation elements. The distribution of Fe, Ni and Cr was uniform without segregation. Figure 8 indicates that vanadium is uniformly distributed in the No. 3 alloy without segregation, while the segregation region of other elements does not change significantly. The solubility of vanadium in austenite is high, and the formation of V (C, N) will affect the microstructure and properties of the alloy [30]. Fine carbides are formed at grain boundaries and the pinning action hinders the growth of dendrites. This will reduce segregation and cause it to be more uniform [31].

**Figure 9:**
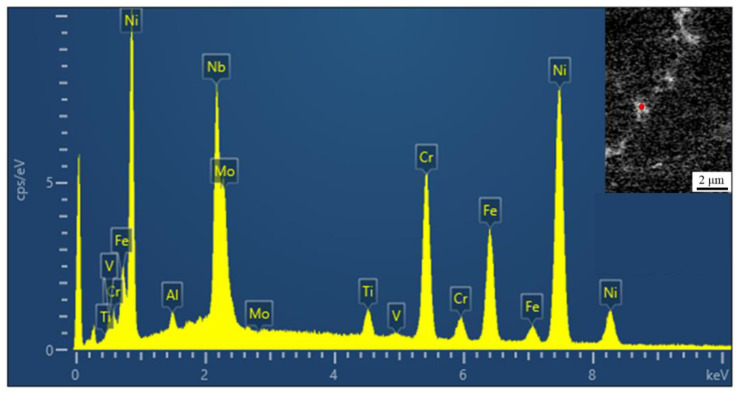
The EDS analysis of the Laves phase in No.3 alloy.

The microstructural evolution of the IN718 alloy in the molten pool can be depicted as follows: L → L + γ → (L + NbC/γ) → L + Laves/γ. Although γ + NbC eutectic can also form during solidification, the amount of γ + NbC eutectic is negligible compared to that of γ + Laves eutectic due to the very low carbon content in the IN718 alloy. Figure 9 shows an EDS point analysis of the Laves phase, and the insert image shows the typical block Laves phase appearance. Nickel has the highest peak value among the elements, followed by niobium. The illustration verifies that the segregation of niobium is mainly concentrated between the dendrites. It is known that the addition of vanadium will improve element segregation. The chemical compositions of the Laves phase of the different alloys are listed in Table 3. It can be seen from Table 3 that the niobium content of the Laves phase in the samples with vanadium added decreases, indicating that the addition of vanadium will improve element segregation. The niobium content of the No. 2 alloy, No. 3 alloy and No. 6 alloy is less than 20%. It can be predicted that the difficulty of homogenization treatment of these alloys is reduced compared with others.

### 3.3. Effect of Vanadium Content on Mechanical Properties of Samples

This part of the mechanical properties, including hardness, residual stress, and tensile properties, are compared for different test alloys. Hardness, as a preliminary assessment of the wear resistance of an alloy, is used to measure the degree of hardness and softness of the material [32]. The distribution of the segregation phase exerts an influence on the difference in hardness of the IN718 alloy, and it is necessary to measure the Vickers hardness on different tested alloys [33,34].

**Figure 10:**
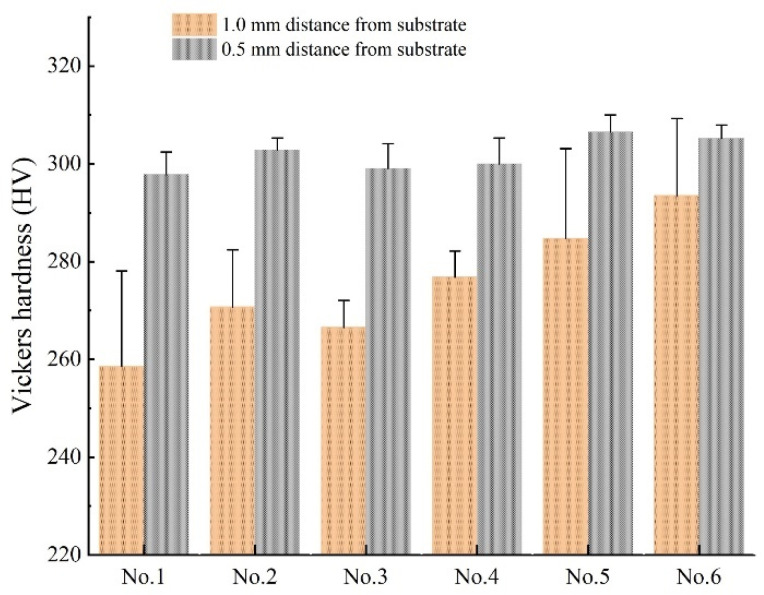
Comparison of Vickers hardness between tested alloys with different locations.

The hardness of two different positions was tested for various test alloys, as shown in Figure 10. Through the comparison of different positions from the substrate, it can be found that the hardness of the position far from the substrate is higher than the hardness of the position near the substrate for all the tested alloys. This trend is consistent for the IN718 alloy, as shown in reference [33]. In addition, the Vickers hardness of the vanadium-added alloy is higher than that of the No. 1 alloy of the blank sample, which is due to the influence of the distribution of the Laves phase [33,34,35,36]. However, the hardness of the alloy with vanadium added is not an evident trend in different locations, so the most appropriate content of the vanadium element cannot be obtained.

In the laser cladding process, the characteristics of rapid heating and rapid cooling of the molten pool will accumulate residual stress, which is regarded as the recrystallization power of the test alloys [37]. Similarly, as part of the mechanical properties, the residual stress was measured by the Vickers micro-indentation method [37,38]. The measured point is consistent with the microhardness point. It can be assumed that the residual stress is in an equi-biaxial state, and the uniaxial stress–strain curve obeys a power-law function. In addition, the residual stress and residual strain are found to conform to the relationship shown in the following equation [17,18]:

(1) $$H=C\sigma \left(\varepsilon_{\mathrm{repr}}+\varepsilon_{\mathrm{res}}\right)$$

(2) $$c^{2}=c_{0}^{2}-0.32\ln\left[1+\frac{\sigma_{\mathrm{res}}}{\sigma \left(\varepsilon_{\mathrm{res}}\right)}\right]$$

where *c*^2^ is recorded as the indentation area ratio, defined as *c*^2^ = A_0_/A_nom_, A_0_ is the actual contact area between the indenter and the test alloys, and A_nom_ is the nominal contact area. The schematic diagram of this part is shown in Figure 8a. H is the hardness value of the test point, and C is a constant equal to 3 [39]. $\sigma \left(\varepsilon_{\mathrm{repr}}+\varepsilon_{\mathrm{res}}\right)$ is the von Mises value obtained when the equivalent strain is $\varepsilon_{\mathrm{repr}}+\varepsilon_{\mathrm{res}}$, and the value of $\varepsilon_{\mathrm{repr}}$ is 0.08. $\varepsilon_{\mathrm{res}}$ is the absolute value of the equivalent residual plastic strain of the von Mises value, and $c_{0}^{2}$ is the corresponding ratio under the condition of no stress, which is taken as 1. Since the tested alloy is directly deposited without heat treatment, the following stress–strain relationship is used [37]:

(3) $$\sigma \left(\varepsilon_{\mathrm{res}}\right)=1181.21\varepsilon_{p}^{0.1754}$$

The previous three equations are simplified, and the expressions of residual strain and residual stress of the test alloys are obtained:

(4) $$\varepsilon_{\mathrm{res}}={\left(\frac{H}{3\times 1181.21}\right)}^{\frac{1}{0.1754}}-0.08$$

(5) $$\sigma_{\mathrm{res}}=1181.21\times {\left|\varepsilon_{\mathrm{res}}\right|}^{0.1754}\times \left[\exp\left(\frac{c^{2}-1}{0.32}\right)-1\right]$$

**Figure 11:**
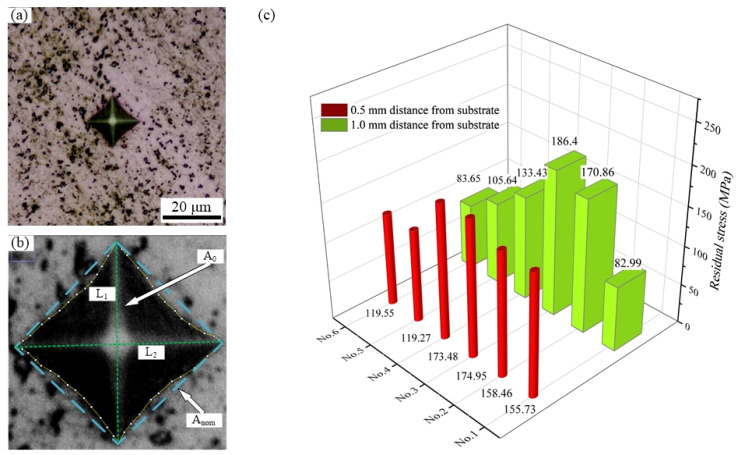
(**a**) Vickers hardness testing, (**b**) schematic of the nominal projected contact area A_nom_, (**c**) comparison of residual stress with different tested alloys.

The measured residual stress values are shown in Figure 11c for different test alloys. It can be observed that the residual stress values of the vanadium-added samples show a certain degree of dispersion. Due to the uneven temperature distribution during the laser cladding process, the thermal stress formed will have a certain range of variation. However, by comparing the residual stress at different positions, it is found that the residual stress value of the alloy with vanadium is higher than that of the blank alloy as a whole, which is caused by the high hardness of the vanadium-added alloy. In addition, it is found that the residual stress of the No. 6 alloy is smaller than that of the No. 1 alloy. This is an interesting phenomenon, and further research is needed. The residual stress of the No. 2 alloy and No. 3 alloy is more pronounced, and it has also been proven that high residual stress will lead to finer grain size and cause their PDS to be smaller than that of the No. 1 alloy [32,37].

**Figure 12:**
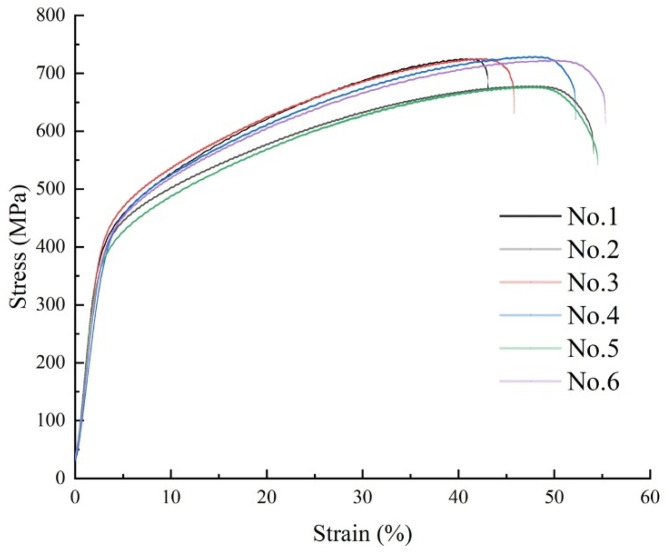
The stress–strain curve of all tested alloys.

Finally, the tensile properties of the IN718 alloy, with different contents of vanadium, were measured at room temperature; these properties included tensile strength (TS), elongation (EL) and yield strength (YS). In order to avoid the influence of material anisotropy on the experimental results, the directions of all samples were the same, as shown in Figure 3. The stress–strain curve is displayed in Figure 12.

In addition, Table 4 shows the tensile properties of all tested alloys at room temperature. It was found that the yield strength and tensile strength of the alloy with the vanadium element were slightly reduced, which was mainly due to the positive effect of the Laves phase on the inhibition of crack propagation [32]. However, the Laves phase is a hard and brittle phase with poor plastic deformation ability [39]. Therefore, the Laves phase content is the highest in the No. 1 alloy, the elongation of which is the lowest among all alloys. In summary, it can be established that, among all the tested alloys in this research, the optimal alloy is the No. 2 alloy.

## 4. Conclusions

Different concentrations of vanadium have different effects on IN718 alloy. During the laser cladding process, the addition of vanadium has a positive impact on the microstructure, segregation and mechanical properties of IN718 alloy. The following conclusions are obtained:

1. The addition of vanadium has little effect on the volume fraction of the dendrite core in the IN718 alloy, but the PDS of the alloys can be significantly reduced, and the values of the No. 2 alloy and No. 3 alloy are the smallest.
2. With the addition of vanadium, the porosity of the cladding layer has been significantly reduced. Analysis of the element distribution in the cladding layer shows that no segregation of the V element occurs, and the main segregated elements are Mo and Nb. The addition of vanadium will reduce the concentration of Nb in the Laves phase. It can be predicted that the difficulty of homogenization treatment of the No. 2 alloy, No. 3 alloy and No. 6 alloy is reduced compared with others.
3. The Vickers hardness value of the cladding layer is measured. The addition of vanadium to IN718 alloy will increase its hardness, which also indicates that the wear resistance of the cladding layer after the addition of the vanadium element will increase. Using the Vickers hardness indentation method to measure the residual stress of the cladding layer, it is found that the addition of vanadium has a negative effect on residual stress, and that the residual stresses of the No. 2 alloy and No. 3 alloy are the largest.
4. The addition of vanadium powder can significantly enhance the elongation of IN718 alloy, but it has a slightly negative effect on yield strength and tensile strength. In summary, the IN718 alloy with a vanadium content of 0.081 wt.% has a comprehensive microstructure and mechanical properties.

## Figures and Tables

**Table 1:** Chemical composition of the master IN 718 alloy (weight percentage, wt.%).

Elements	No.1	No.2	No.3	No.4	No.5	No.6
V	/	0.081	0.18	0.45	1.06	1.88

**Table 2:** Processing parameters of laser cladding [20].

Parameters	Laser Power (W)	Scanning Speed (mm/s)	Powder Federate (g·min^−1^)	Z-Increment (mm)
-	1200	8	14	0.7

**Table 3:** Chemical compositions of the Laves phase in different alloys (wt.%).

Alloy	Al	Ti	Cr	Fe	Ni	Nb	Mo
No.1	0.02	2.24	13.18	16.98	34.97	26.15	6.28
No.2	0.51	1.45	14.96	13.55	45.21	19.17	5.12
No.3	0.82	1.76	14.31	13.17	46.92	18.40	4.61
No.4	0.50	1.45	14.46	13.16	44.40	20.94	4.94
No.5	0.57	1.55	14.92	13.66	43.73	21.19	3.98
No.6	0.61	1.54	14.44	12.94	46.42	18.95	4.59

**Table 4:** Room-temperature tensile properties of all tested alloys.

Alloys	No.1	No.2	No.3	No.4	No.5	No.6
Tensile strength (MPa)	725.8 ± 12	678.6 ± 21	725.9 ± 19	729.2 ± 23	677.6 ± 25	722.9 ± 28
Yield strength (MPa)	716.7 ± 30	671.9 ± 27	716.7 ± 32	712.2 ± 28	669.8 ± 33	700.9 ± 38
Elongation (%)	35.5 ± 3.1	51.5 ± 2.7	46.2 ± 3.5	46.9 ± 2.5	47.7 ± 2.1	46.2 ± 3.6

## Data Availability

Data sharing not applicable.
